# Implicit Regularization for Reconstructing 3D Building Rooftop Models Using Airborne LiDAR Data

**DOI:** 10.3390/s17030621

**Published:** 2017-03-19

**Authors:** Jaewook Jung, Yoonseok Jwa, Gunho Sohn

**Affiliations:** Department of Earth and Space Science and Engineering, York University, 4700 Keele Street, Toronto M3J 1P3, ON, Canada; jwjung00@gmail.com (J.J.); yjwa@yorku.ca (Y.J.)

**Keywords:** 3D building rooftop modeling, building reconstruction, regularization, airborne laser scanning data, minimum description length

## Abstract

With rapid urbanization, highly accurate and semantically rich virtualization of building assets in 3D become more critical for supporting various applications, including urban planning, emergency response and location-based services. Many research efforts have been conducted to automatically reconstruct building models at city-scale from remotely sensed data. However, developing a fully-automated photogrammetric computer vision system enabling the massive generation of highly accurate building models still remains a challenging task. One the most challenging task for 3D building model reconstruction is to regularize the noises introduced in the boundary of building object retrieved from a raw data with lack of knowledge on its true shape. This paper proposes a data-driven modeling approach to reconstruct 3D rooftop models at city-scale from airborne laser scanning (ALS) data. The focus of the proposed method is to implicitly derive the shape regularity of 3D building rooftops from given noisy information of building boundary in a progressive manner. This study covers a full chain of 3D building modeling from low level processing to realistic 3D building rooftop modeling. In the element clustering step, building-labeled point clouds are clustered into homogeneous groups by applying height similarity and plane similarity. Based on segmented clusters, linear modeling cues including outer boundaries, intersection lines, and step lines are extracted. Topology elements among the modeling cues are recovered by the Binary Space Partitioning (BSP) technique. The regularity of the building rooftop model is achieved by an implicit regularization process in the framework of Minimum Description Length (MDL) combined with Hypothesize and Test (HAT). The parameters governing the MDL optimization are automatically estimated based on Min-Max optimization and Entropy-based weighting method. The performance of the proposed method is tested over the International Society for Photogrammetry and Remote Sensing (ISPRS) benchmark datasets. The results show that the proposed method can robustly produce accurate regularized 3D building rooftop models.

## 1. Introduction

A key problem domain that we address in this paper is to reconstruct a 3D geometric model of building rooftop from remotely sensed data such as airborne laser point clouds. The representation that we follow for 3D rooftop models draws on ideas from geometric modeling used in Photogrammetry and Geographical Information Science (GIS). In the representation scheme, a 3D rooftop is modeled with either primitive geometric elements (i.e., points, lines, planes and objects), or primitive topological elements (i.e., vertices, edges, faces, and edge-groups (rings of edges on faces)). Typically, both primitive geometric and topological elements are used together for representing 3D rooftop models (e.g., CityGML and Esri ArcGIS’s shapefile). CityGML is an open data model and XML-based format for the storage and exchange of virtual 3D city models [1].

In CityGML, 3D rooftop models can be differently represented according to the level-of-detail (LoD). A prismatic model of rooftop that is a height extrusion of a building footprint is defined as LoD1 in CityGML, while LoD2 requires a detailed representation of the primitive geometric and topological elements in a 3D rooftop model. An important aspect in GIS-driven 3D model representation is that the reconstructed model elements should correspond to semantically meaningful spatial entities used in architecture, civil and urban planning: for instance, the reconstructed geometric elements represent roof lines (ridges and eaves), roof planes (gables and hips), vents, windows, doors, wall columns, chimneys, etc. Thus, a photo-realistic reconstructed rooftop model can be used for assisting human decisions on but not limited to asset management, renovating planning, energy consumption, evacuation planning, etc. As discussed in Rottensteiner et al. [2], a city-scale building model will provide an important mean to manage urban infrastructure more effectively and safely for addressing critical issues related to rapid urbanization. In this study, we aim to reconstruct LoD2 models of the rooftops from remote sensed data.

Traditionally, 3D rooftop models are derived through interaction with a user using photogrammetric workstations (e.g., multiple-view plotting or mono-plotting technology). This labor-intensive model generation process is tedious and time-consuming, which is not suitable for reconstructing rooftop models at city-scale. As an alternative method, great research efforts have been made for developing a machine-intelligent algorithm to reconstruct photo-realistic rooftop models in a fully-automated manner for the last two decades [3]. Recently, airborne light detection and ranging (LiDAR) scanners became one of the primary data acquisition tools, which enable rapid capturing of targeted environments in 3D with high density and accuracy. Due to these advantages, state-of-the-art technologies for automatically reconstructing 3D rooftop models using airborne LiDAR data have been proposed by many researchers [2,3,4,5,6]. However, only limited success in a controlled environment has been reported, and the success of developing an error-free rooftop modeling algorithm is not achieved yet [2].

In general, 3D rooftop models are derived automatically from 3D LiDAR point clouds by: (1) extracting the primitive geometric elements, namely “modeling cues”; and (2) recovering the primitive topological elements among the modeling cues. A critical problem to hinder the automation of 3D rooftop model generation is that many portions of the object (rooftop) are unknown, and recovered with errors caused by the following reasons:

- Irregular point distribution: Despite the advantages of acquiring highly accurate and dense 3D point clouds over rooftops by airborne LiDAR, the sensor also has its limitations. Airborne LiDAR transmits a packet of collimated laser beams through an electro-optical scanner, and computes a location of scatter, which surface is reflected from the transmitted laser energy, by measuring a range between the transmitter and scatter with known position and orientation of the laser scanner. The size of the beam footprint and space between adjacent laser points on the ground are determined by the flying height of the airborne platform and scanning frequency. In addition, the weak energy reflectance due to absorption and ill-posed surface angle against scanning pose, where the peak is below a pre-defined threshold, are discarded. Thus, all these system variables produce an irregular distribution of laser point clouds over the targeted object surface. Consequently, the modeling cues are often generated with errors, or are fragmented, or completely missing. These errors have a negative impact on the derivation of the topological elements, and thus the accuracy of rooftop model generation.
- Occlusion: Similar to other sensors, airborne LiDAR also suffers from difficulties in capturing a complete view of objects due to occlusions. A disadvantageous viewing angle between the laser beam direction and object pose may hinder the illumination of laser beams on certain object surfaces, where no laser points are generated. In theory, airborne LiDAR has an ability to penetrate foliage; however, the amount of returned laser energy varies depending on tree species, their maturity, seasonal effect and relative viewing angle between the laser beam and the leaf surface angle. A weak reflected energy will be neglected and not be able to produce any laser points over certain areas of roofs where tree grows nearby. In addition, in urban area, buildings are occluded by adjacent buildings which are found in the path between the sensor and the surface to survey. These negative effects cause errors in recovering the primitive topological elements for reconstructing the rooftop model.
- Unreliable data analysis: A few of the laser point cloud analytics are applied to detecting building objects, classifying non-roof-related objects (e.g., trees, roof superstructures, etc.), segmenting roof planar patches, extracting corners and line primitives, and other algorithms related to recovering the primitive topological elements (e.g., boundary tracing, edge-linking, etc.). The performance of these algorithms varies depending on data resolution, scene complexity and noise; they may produce some errors, which has a negative effect on recovering both modeling cues and topological elements.

As discussed previously, the aforementioned factors lead to errors in recovering the modeling cues sufficiently well for generating an error-free rooftop model. Typically, knowledge of a rooftop object of interest (e.g., roof type, structure, number of roof planes, etc.) is unknown. Thus, recovering all the primitive topological elements accurately with an error-free geometric model is a very challenging vision task. To address this issue, many researchers have introduced some modeling constraints to compensate the limitations of erroneous modeling cues [7,8,9,10]. These constraints are used as prior knowledge on targeted rooftop structures: (1) for constructing the modeling cues to conform to Gestalt law (i.e., parallelism, symmetry, and orthogonality), and linking fragmented modeling cues in the frame of perceptual grouping; and (2) by determining optimal parametric rooftop model fit into part of rooftop objects through a trial-and-error of model section from a given primitive model database. We refer these modeling constraints as an “explicit regularity” imposed on rooftop shape as the definition of regularity is fully and clearly described. However, only a few of the explicit regularity terms can be applicable, and the shapes of rooftops in reality appear too complex to be reconstructed with those limited constraints.

In this paper, we focus on the data-driven modeling approach to reconstruct 3D rooftop models from airborne LiDAR data by introducing flexible regularity constraints that can be adjusted to given objects in the recovery of modeling cues and topological elements. The regularity terms that are used in this study represent a regular pattern of the line orientations, and the linkage between adjacent lines. In contrast to the term of “explicit regularity”, we refer to it as an “implicit regularity” because its pattern is not directly expressed, but found with given data and object (rooftop). This implicit regularity is used as a constraint for changing the geometric properties of the modeling cues and topological relations among adjacent modeling cues to conform to a regular pattern found in the given data. This data-adaptive regularity (or regularization process) allows us to reconstruct more complex rooftop models.

In this paper, we describe a pipeline of 3D rooftop model reconstruction from airborne LiDAR data. First, to gain some computational efficiency, we decompose a rooftop object into a set of homogeneous point clouds based on height similarity and plane similarity, from which the modeling cues of line and plane primitives are extracted. Secondly, the topological elements among the modeling cues are recovered by iteratively partitioning and merging over a given point space with line primitives extracted at a global scale using the Binary Space Partitioning (BSP) technique. Thirdly, errors in the modeling cues and topological elements are implicitly regularized by removing erroneous vertices or rectifying the geometric properties to conform to globally derived regularity. This implicit regularization process is implemented in the framework of Minimum Description Length (MDL) combined with Hypothesize and Test (HAT). The parameters governing the MDL optimization are automatically estimated based on Min-Max optimization and Entropy-based weighting method. The proposed parameter estimators provide optimal weight values that adapt according to building properties such as; size, shape, and the number of boundary points. The proposed pipeline of rooftop model generation was developed based on previous works reported in [11]. We extended the work by proposing data-adaptive parameter estimation, conducting an extensive performance evaluation and engineering works to implement a computationally efficient modeling pipeline.

### Related Works

Numerous building reconstruction algorithms have been published for the past two decades. Although it is difficult to clearly classify these various methods into specific categories, there are several ways to categorize the methods: the used data source (single vs. multi-sources), the data processing strategy (data-driven (or generic), model-driven (or parametric)), and the amount of human interaction (manual, semi-automatic, or fully automated) [12]. Of those, classifying existing methods into data-driven or model-driven approaches provides a good insight for understanding and developing 3D building model reconstruction algorithms.

In the model-driven approaches, 3D building models are reconstructed by fitting parameterized primitives to data. This is possible because many buildings in rural and suburban area have common shapes in whole building or building roof parts. These common roof shapes such as flat, gable, and hip roof are considered as standard primitives for representing building rooftop structures. Simple buildings can be well represented as regularized building models using pre-defined parameterized primitives even with low density data and presence of missing data. However, complex buildings and arbitrarily shaped buildings are difficult to model using a basic set of primitives. In addition, the selection of the proper primitives among a set of primitives is not an easy task. To address the limitations, Verma et al. [8] presented a parametric modeling method to reconstruct relatively complex buildings by combining simple parametric roof shapes that are categorized into four types of simple primitives. In this study, the roof-topology graph is constructed to represent the relationships among the various planar patches of approximate roof geometry. The constructed roof-topology graph is decomposed into sub-graphs, which represents simple parametric roof shapes, and then parameters of the primitives are determined by fitting LiDAR data. Although they decomposed complex buildings into simple building parts, many building parts cannot be still explained by their four simple shape primitives. Similarly, Milde et al. [13] reconstructed 3D building models by matching sub-graphs of the region adjacency graph (RAG) with five basic roof shapes and then by combining them using three connectors. Kada and McKinley [14] decomposed the building’s footprint into cells, which provided the basic building blocks. Three types of roof shapes including basic, connecting, and manual shapes are defined. Basic shapes consist of flat, shed, gabled, hipped, and Berliner roofs while connecting shapes are used to connect the roofs of the sections with specific junction shapes. The parameterized roof shapes of all cells are determined from the normal direction of LiDAR points. The entire 3D building model is represented by integrating the parameterized roof elements with the neighboring pieces. Although a high level of automation is achieved, the method still requires manual works to adjust cell parameters and to model more complex roof shapes like mansard, cupola, barrel, and even some detail elements. Lafarge et al. [15] reconstructed building models from a digital surface model (DSM) by combining generic and parametric methods. Buildings are considered as assemblages of 3D parametric blocks from a library. After extracting 2D building supports, 3D parametric blocks are placed on the 2D supports using Gibbs model, which controls both the block assemblage and the fitting to data. The optimal configuration of 3D blocks is determined using the Bayesian framework. They mentioned that the optimization step needs to be improved to achieve both higher precision and shorter computing time as future work. Based on a predefined primitive library, Huang et al. [10] conducted a generative modeling to reconstruct roof models that fit the data. The library provides three groups including 11 types of roof primitives whose parameters consist of position parameters, contour parameters, and shape parameters. Building roofs are represented as one primitive or an assemblage of primitives allowing primitives overlaps. For combining primitives, they derived combination and merging rules which consider both vertical and horizontal intersections. Reversible Jump Markov Chain Monte Carlo (RJMCMC) with a specified jump mechanism is conducted for the selection of roof primitives, and the sampling of their parameters. Although they have shown potential and flexibility of their method, there are issues to be solved: (1) uncertainty and instability of the reconstructed building model; (2) influence of prior knowledge and scene complexity on completeness of the reconstruction; and (3) heavy computation time.

In contrast with model-driven approaches, data-driven approaches do not make any assumptions regarding to the building shapes, thus they can theoretically handle all kinds of buildings. However, the approach may cause considerable deformations due to the sensitivity to surface fluctuations and outliers in the data. In addition, it requires a regularization step during the reconstruction process. In general, the generic approach starts by extracting building modeling cues such as surface primitives, step lines, intersection lines, and outer boundary lines followed by reconstructing the 3D building model. The segmentation procedure for extracting surface primitives divides a given data set into homogeneous regions. Classical segmentation algorithms such as region growing [16,17] and RANSAC [18] can be used for segmenting building roof planes. In addition, Sampath and Shan [19] conducted eigenanalysis for each roof point within its Voronoi neighborhood, and then adopted the fuzzy k-means approach to cluster the planar points into roof segments based on their surface normal. Then, they separated the clusters into parallel and coplanar segments based on their distance and connectivity. Lafarge and Mallet [20] extracted geometric shapes such as planes, cylinders, spheres, or cones for identifying the roof sections by fitting points into various geometric shapes, and then proposed a method for arranging both the geometric shapes and the other urban components by propagating point labels based on MRF. Yan et al. [21] proposed a global solution for roof segmentation. Initial segmentation is optimized by minimizing a global energy function consisting of the distances of LiDAR points to initial planes, spatial smoothness between data points, and the number of planes. After segmenting points or extracting homogeneous surface primitives, modeling cues such as intersection lines and step lines can be extracted based on geometrical and topological relationships of the segmented roof planes. Intersection lines are easily obtained by intersecting two adjacent planes or segmented points while step lines are extracted at roof plane boundary with abrupt height discontinuity. To extract step lines, Rottensteiner et al. [16] detected edge candidate points and then extracted step lines from an adjustment considering edge points within user-specified threshold. In addition, Sohn et al. [22] proposed a step line extractor, called Compass Line filter (CLF), for extracting straight lines from irregularly distributed LiDAR points. Although outer boundary is one type of step line, it is recognized as a separate process in many data-driven approaches. Some researchers delineated initial boundary lines from building boundary points using alpha shape [23], ball-pivoting [8], and contouring algorithm [24]. Then, the initial boundary was simplified or regularized. The detail reviews for simplification or regularization of boundary will be given in following paragraphs. Once all building modeling cues are collected, 3D building models are reconstructed by aggregating the modeling cues. To reconstruct topologically and geometrically correct 3D building models, Sohn et al. [22] proposed the BSP technique, which progressively partitions a building region into homogeneous binary convex polygons. Rau and Lin [25] proposed a line-based roof model reconstruction algorithm, namely TIN-Merging and Reshaping (TMR), to reconstruct topology with geometric modeling. Oude Elberink and Vosselman [26], and Perera and Maas [27] used a roof topology graph to preserve roof topology. In the latter, roof corners are geometrically modeled using the shortest closed cycles and the outermost cycle derived from the roof topology graph.

Detection of building boundary is an intermediate step for 3D building reconstruction although it is not required in all building reconstruction algorithms. Generally, the initial boundaries extracted from irregular LiDAR points have jagged shape with large numbers of vertices. Thus, a simplification or regularization process is required to delineate plausible building boundaries with certain regularities such as orthogonality, parallelism, and symmetry. Various techniques related to the regularization of building boundary have been proposed in the literature [28]. In most methods, the boundary detection process starts by extracting boundary points from segmented points. From extracted boundary points, initial building boundaries are generated by tracing boundary points followed by a simplification or regularization process, which improves the initial boundary. The easiest method to improve initial boundary is to simplify the initial boundary by removing vertices but preserving relevant points. The well-known Douglas–Peucker (DP) algorithm [29] is widely recognized as the most visually effective line simplification algorithm. The algorithm starts by selecting two points which have the longest distance and recursively adding vertices whose distance from the line is less than a given threshold. However, the performance of the algorithm fully depends on the used threshold and is substantially affected by outliers. Another approach extracts straight lines from boundary points using the Hough Transform [30] or using RANSAC [31]. The extracted lines are then connected by intersections of the extracted straight lines to generate closed outer boundary lines. However, Brenner [28] pointed out that the methods require some additional steps due to missing small building edges.

On the other hand, the regularization process imposes certain regularities when the initial boundary is simplified. Vosselman [7] assumed that building outlines are along or perpendicular to the main direction of a building. After defining the position of a line by the first two boundary points, the line is updated using the succeeding boundary points until the distance of a point to the line exceeds some bound. The next line starts from this point in a direction perpendicular to the previous line. A similar approach was proposed by Sampath and Shan [9]. They grouped points on consecutive edges with similar slopes and then applied a hierarchical least squares solution to fit parametric lines representing the building boundary.

Some methods are based on the model hypothesis and verification approach. Ameri [32] introduced the Feature Based Model Verification (FBMV) for modification and refinement of polyhedral-like building objects. In their approach, they imposed the geometrical and topological model information to the FBMV process as external and internal constraints which consider linearity for straightening consecutive lines, connectivity for establishing topology between adjacent lines, orthogonality, and co-planarity. Then, the weighted least squares minimization was adopted to produce a good regularized description of a building model. Weidner and Förstner [33] adopted the MDL concept to regularize noisy building boundaries. For four local consecutive points, ten different hypothetical models are generated with respect to regularization criteria. Then, MDL, which depends on the mutual fit of the data and model and on the complexity of the model, is used to find the optimal regularity of the local configuration. Jwa et al. [34] extended the MDL-based regularization method by proposing new implicit hypothesis generation rules and by re-designing model complexity terms where line directionality, inner angle and number of vertices are considered as geometric parameters. Furthermore, Sohn et al. [11] used the MDL-based concept to regularize topologies within rooftop model. Zhou and Neumann [35] introduced global regularities in building modeling to reflect the orientation and placement similarities among 2.5D elements, which consist of planar roof patches and roof boundary segments. In their method, roof-roof regularities, roof-boundary regularities, and boundary-boundary regularities are defined and then the regularities are integrated into a unified framework.

## 2. 3D Building Rooftop Reconstruction

**Figure 1:**
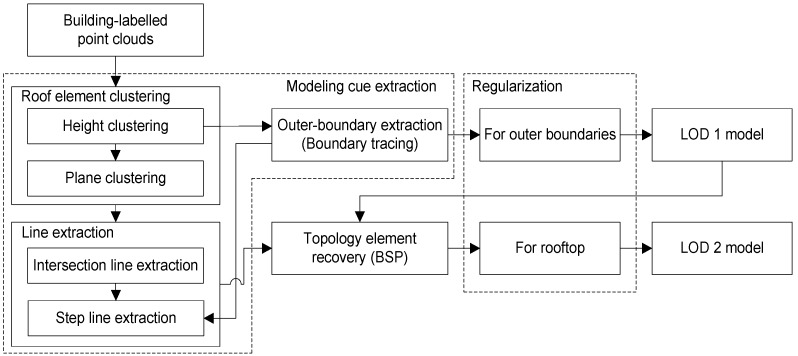
The overall workflow developed for reconstructing 3D rooftop models.

Figure 1 shows the overall workflow implemented for generating 3D building rooftop models from airborne LiDAR point clouds, where individual buildings are detected. The method consists of three main parts: (1) modeling cue extraction; (2) topology element reconstruction; and (3) regularization. In the modeling cue extraction, roof element clusters, lines (intersection and step lines), and outer-boundaries are extracted from a set of laser point clouds labeled as single building objects (i.e., building labeled points) (Section 2.1). Then, the topology relations among the modeling cues are established by BSP (Section 2.2). Finally, an implicit regularization process is applied to outer-building boundaries and rooftop polygons (Section 3). The regularization process is based on the framework of MDL in combination with HAT optimization. Note that the regularization process is conducted twice; once for regularizing building outer-boundaries which represent LoD1 models, and then for rooftop models which represent LoD2 models. Two types of weight parameters in the MDL-based objective function are automatically determined by Min-Max optimization and Entropy-based parameter estimation method, respectively (Section 4).

### 2.1. Modeling Cue Extraction

The first step towards generating 3D building models using LiDAR data is to gather the evidence of building structures (i.e., primitive geometric elements). Planes and lines are recognized as the most important evidence to interpret building structures due to the fact that 3D building rooftop models can be mainly represented by planar roof faces and edges. The two different modeling cues (planar and linear modeling cues) have different properties and can be separately extracted from LiDAR points. In Section 2.1.1, building points are sequentially segmented into homogeneous clusters, first based on height similarity and then based on plane similarity. In Section 2.1.2, linear modeling cues are extracted using boundary points of the homogeneous clusters.

#### 2.1.1. Roof Element Clustering

Roof element clustering segments building-labeled points into homogeneous rooftop regions with a hierarchical structure. A building rooftop in an urban area is a combination of multiple stories, each of which consists of various shapes of flat and sloped planes. Directly extracting homogeneous regions from entire building points may result in difficulties due to a high degree of shape complexity. To reduce the complexity, the building-labeled points are decomposed into homogeneous clusters by sequentially applying height similarity and plane similarity in order.

In the height clustering step, the rooftop region $R=\left\{p_{i}|i=1,\;2,\ldots ,n\right\}$ with *n* numbers of building-labeled points is divided into *m* height clusters $R=\left\{S_{1},\;S_{2},\ldots ,S_{m}\right\}$. Height similarity at each point is measured over its adjacent neighboring points in Triangulated Irregular Network (TIN). A point with the maximum height is first selected as a seed point, and then a conventional region growing algorithm is applied to add neighbor points to a corresponding height cluster with a certain threshold ($\delta_{h}=1\;m$). This process is repeated until all building rooftop points are assigned to one of the height clusters. As a result, the height clusters satisfy the property $R=\cup_{i=1}^{M}S_{i}$, $S_{i}\cap S_{j}=\{\}$, ∀i≠j. Note that each height cluster consists of one or more different roof planes.

**Figure 2:**
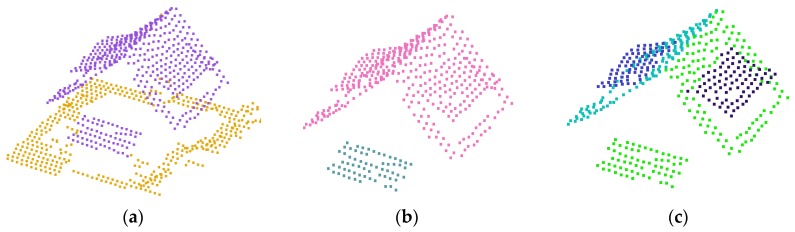
Roof element clustering: (**a**) building-labeled points (purple); (**b**) height clustering (pink and green); and (**c**) plane clustering (black, pink, blue and purple).

In the plane clustering step, each height cluster is decomposed into *k* plane clusters $\Pi =\left\{\pi_{1},\pi_{2},\ldots ,\pi_{k}\right\}$ based on a plane similarity criterion. The well-known random sample consensus (RANSAC) algorithm is adopted to obtain reliable plane clusters as suggested in previous studies [18,36]. The process starts by randomly selecting three points as seed points to generate an initial plane. After a certain period of random sampling, a plane, which has the maximum number of inliers with a user defined tolerance distance *ζ* (*ζ =* 0.1 m) from the estimated plane, is selected as a best plane. Points, which are assigned in the previous iteration, are excluded in the next step. The process continues until all points of the height cluster are assigned into certain plane clusters. Figure 2b,c shows examples of height clusters and plane clusters, respectively, where different colors represent different clusters.

#### 2.1.2. Linear Modeling Cue Extraction

Once building-labeled points are segmented into homogeneous clusters with a hierarchical structure, linear modeling cues are extracted from the homogeneous clusters. We divide linear modeling cues into three different types in order to reduce the complexity in the modeling cue extraction process as follows: (1) outer boundaries of height clusters; (2) intersection lines; and (3) step lines within each height cluster.

In boundaries of height clusters, two adjacent planes have a large height discontinuity. Thus, outer boundaries of height clusters can be recognized as step lines. However, distinguishing between outer boundaries of height clusters and step lines within each height cluster can reduce ambiguity in the topology recovering process (Section 2.2). In addition, outer boundaries of height clusters can serve to generate the LoD1 model. For these reasons, in this study, we separately extract outer boundaries of height clusters. The process starts by detecting boundary points of height clusters which share neighbor height clusters in a TIN structure. After selecting a starting boundary point, a next boundary point is determined by surveying neighbor boundary points, which are connected with the previous boundary point in TIN structure, and by selecting a boundary point which appears first in an anti-clockwise direction. The process continues until the boundary is closed. Then, the closed boundary is regularized by the MDL-based regularization method which will be described in Section 3.

An intersection line candidate is extracted by two adjacent roof planes. Candidates are accepted as valid intersection line if they separate the point sets of the planes and if a sufficient number of points is close to the generated lines.

**Figure 3:**
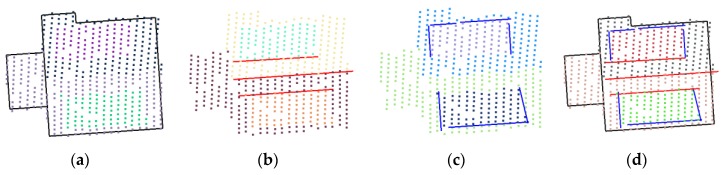
Modeling cues extraction: (**a**) outer boundaries (black); (**b**) intersection lines (red); (**c**) step lines (blue); and (**d**) combined modeling cues.

For step lines, boundary points of plane clusters, which do not belong to outer boundaries or intersection lines, are considered as candidate points for step lines. Given a sequence $D=\left\{c_{1},\;c_{2},\ldots ,c_{l}\right\}$ of *l* candidate points, step lines are extracted in a similar way to the Douglas–Peucker (DP) algorithm. The process starts with a straight line ($\bar{c_{1}c_{l}}$) connecting the first point and last point of the sequence and then recursively adding candidate points which have a distance larger than a user-defined tolerance (0.5 m). Each segment of the line segments is considered a step line. Figure 3 gives examples of each type of linear modeling cues.

### 2.2. BSP-Based Topology Construction

**Figure 4:**
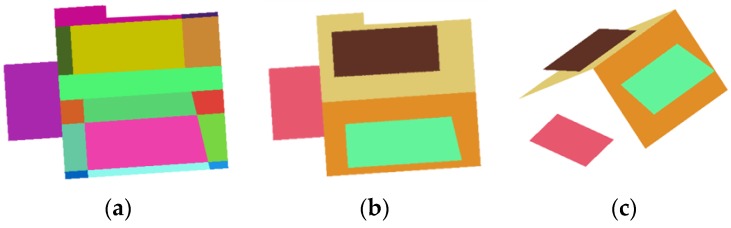
Binary Space Partitioning: (**a**) partitioning step; (**b**) merging step; and (**c**) reconstructed model.

Once all modeling cues are collected, topological relations among the modeling cues are constructed by the BSP technique. In computer science, the BSP is a hierarchical partitioning method for recursively subdividing a space into convex sets with hyperlines. Sohn et al. [22] used the BSP to recover topological relations of 3D building rooftop planes. We adopt the method to reconstruct a topologically and geometrically correct 3D building rooftop model from incomplete modeling cues. The topology recovery process consists of a partitioning step and plane merging step. In the partitioning step, a hierarchical binary tree is generated by dividing a parent region into two child regions with hyperlines (linear modeling cue). The partitioning optimum is achieved by maximizing partitioning score which consists of planar homogeneity, geometric regularity and edge correspondence [22]. In plane merging step, the adjacent roof planes having similar normal vector angles are merged by applying a user-defined threshold. The merging process continues until no plane can be accepted by the co-planar similarity test. Once all polygons are merged together, 3D building rooftop model can be reconstructed by collecting final leave nodes in the BSP tree. Figure 4 shows results of partitioning step, merging step and corresponding 3D rooftop model.

## 3. Implicit Regularization of Building Rooftop Models

As mentioned before, recovering error-free 3D rooftop models from erroneous modeling cues is a challenging task. Geometric constraints such as parallelism, symmetry, and orthogonality can be explicitly used as a prior knowledge on rooftop structures to compensate the limitations of erroneous modeling cues. However, explicitly imposing the constraints has limitations on describing complex buildings that appear in reality. In this study, we propose an implicit regularization where regular patterns of building structures are not directly expressed, but implicitly imposed on reconstructed building models providing flexibility for describing more complex rooftop models. The proposed regularization process is conducted based on HAT optimization in MDL framework. Possible hypotheses are generated by incorporating regular patterns that are present in the given data. MDL is used as a criterion for selecting an optimal model out of the possible hypotheses. The MDL concept for model selection is introduced in Section 3.1 while Section 3.2 introduces a method for hypothesis generation.

### 3.1. MDL Principle and Rooftop Modeling

The MDL proposed by Rissanen [37] is a method for inductive inference that provides a generic solution to the model selection problem [38]. The MDL is based on the idea of transmitting data as a coded message, where the coding is based on some prearranged set of parametric statistical model. The full transmission has to include not only the encoded data values, but also the coded model parameter values [39]. Thus, the MDL consists of model complexity and model closeness as follows:

(1) DL=λℒ(D|H)+(1−λ)ℒ(H)

where ℒ(D|H) indicates a goodness-of-fit of observations *D* given a model *H* while ℒ(H) represents how complex the model *H* is. λ is a weight parameter for balancing the model closeness and the model complexity. Assuming that an optimal model representing the data has the minimal description length, the model selection process allows a model *H* to be converged to the optimal model H* as follows:

(2) $$H^{*}=arg\;min_{H\in \Phi }\left\{\lambda ℒ\left(D|H\right)+\left(1-\lambda \right)ℒ\left(H\right)\right\}$$

The first term in Equation (1) is optimized for good data attachment to the corresponding model. With an assumption that an irregular distribution of data $D=\left\{x_{1},\ldots ,x_{n}\right\}$ with *n* measurements caused by random errors follows a Gaussian distribution $x\sim N\left(\mu ,\sigma^{2}\right)$ with expectation μ and variance $\sigma^{2}$, its density function can be represented as $P\left(x\right)=\frac{1}{\sigma \sqrt{2\pi }}e^{-\frac{{\left(x-\mu \right)}^{2}}{2\sigma^{2}}}$. By using a statistical model of the data, the degree of fit between a model and data can be measured by $ℒ\left(D|\mu ,\sigma^{2}\right)$, and then the term of model closeness can be rewritten in a logarithmic form as follows:

(3) $$L\left(D|\mu ,\sigma^{2}\right)=-log_{2}P\left(D\right)=-\left(log_{2}e^{-\frac{\sum {\left(x-\mu \right)}^{2}}{2\sigma^{2}}}+\;nlog_{2}\frac{1}{\sigma \sqrt{2\pi }}\right)=\frac{1}{2ln2}\sum {\left(\frac{x-\mu }{\sigma }\right)}^{2}+nlog_{2}\sigma +\frac{n}{2}log_{2}2\pi$$

In Equation (3), the last two terms can be ignored with an assumption that all the hypotheses have the same σ. Thus, the equation is simplified as follows:

(4) $$ℒ\left(D|H\right)=\;\frac{\Omega }{2ln2}$$

where Ω is the weighted sum of the squared residuals between a model *H* and a set of observations *D*, that is ${\left[D-H\right]}^{T}\left[D-H\right]$ in matrix form.

The second term in Equation (1) is designed to encode the model complexity. In this study, the model complexity is explained by three geometric factors: (1) the number of vertices $N_{v}$; (2) the number of identical line directions $N_{d}$; and (3) the inner angle transition $N_{∠\theta }$. By using the three geometric factors, an optimal model is chosen if its polygon has a small number of vertices and a small number of the identical line directions, and if the inner angle transition is smoother or more orthogonal.

**Figure 5:**
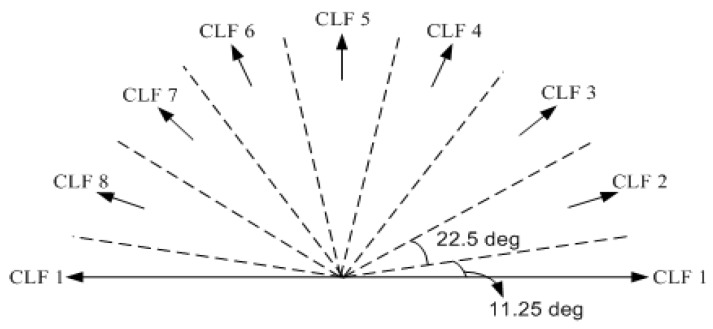
Compass line filter.

Suppose that $N_{v}$, $N_{d}$, and $N_{∠\theta }$ are used for an initial model, while $N_{v}^{\prime }$, $N_{d}^{\prime }$, and $N_{∠\theta }^{\prime }$ are used for a hypothetical model generated from the initial model. To measure the description length for the number of vertices, we start by deriving the probability that a vertex is randomly selected from a given model, $P\left(v\right)=\frac{1}{N_{v}}$. Then, it can be expressed in bits as $log_{2}\left(N_{v}\right)$. Since a hypothetic model generated by hypothesis generation process has $N_{v}^{\prime }$ vertices, its description length is $N_{v}^{\prime }log_{2}\left(N_{v}\right)$. Similarly, the probability for the number of identical line directions $N_{d}$ is $P\left(d\right)=\frac{1}{N_{d}}$ and can be expressed in bits as $log_{2}\left(N_{d}\right)$. By considering the required number of line directions $N_{d}^{\prime }$, the description length for identical line direction is measured by $N_{d}^{\prime }log_{2}\left(N_{d}\right)$. To define line directions, we adopt compass line filter (CLF) suggested by Sohn et al. [22], as shown in Figure 5. The CLF is determined by the whole set of eight filtering lines with different slopes $\left\{\theta_{i}:i=1,\ldots ,8\right\}$ that is equally separated in steps of 22.5°. The representative angle for each slope, $\theta_{i}^{REP}$, is calculated by a weighted averaging of angles that takes the summed line length of each CLF slope into account.

Lastly, the description length for inner angle transition is measured by assigning a certain penalty value to quantized inner angles. As depicted in Equation (5), the penalty values $\gamma_{i=0,1,2}$ are heuristically determined to have the minimum value of 0 (i.e., favour inner angle) if inner angle ∠θ is close to 90° or 180°, while the maximum value of 2 (i.e., unfavorable inner angle) is assigned to very acute inner angles. This is because acute inner angle at two consecutive building vectors rarely appears in reality. Thus, the probability for $N_{∠\theta }$ can be derived from an inner angle that is located in one of the quantized angles, $P\left(∠\theta \right)=\frac{1}{N_{∠\theta }}$, and expressed in bits as $log_{2}\left(N_{∠\theta }\right)$. In the optimal model, the cost imposed by penalty values is $\sum_{k=1}^{N_{v}^{\prime }}\gamma_{i=0,1,2}$, and its description length is calculated by $N_{∠\theta }^{\prime }log_{2}\left(N_{∠\theta }\right)$.

(5) $$\gamma_{i=0,1,2}=\{\begin{array}{cc} 0 & \mathrm{if}\;78.75{}^{\circ}\leq ∠\theta \leq 101.25{}^{\circ}\;\mathrm{or}\;168.75{}^{\circ}\leq ∠\theta \leq 180{}^{\circ}\\ 1 & \mathrm{if}\;11.25{}^{\circ}<∠\theta <78.75{}^{\circ}\;\mathrm{or}\;101.25{}^{\circ}<∠\theta <168.75{}^{\circ}\\ 2 & \mathrm{if}\;0{}^{\circ}\;<∠\theta \leq 11.25{}^{\circ}\; \end{array}$$

As a result, the description length for sub-terms of model complexity ℒ(H) is obtained by the summation of three geometric factors as follows:

(6) $$ℒ\left(H\right)=\;W_{v}N_{v}^{\prime }log_{2}N_{v}+W_{d}N_{d}^{\prime }log_{2}N_{d}+W_{∠\theta }N_{∠\theta }^{\prime }log_{2}N_{∠\theta }$$

where $W_{v}$, $W_{d}$, and $W_{∠\theta }$ are weight values for each sub-factor in the model complexity.

### 3.2. Hypothesis Generation

**Figure 6:**
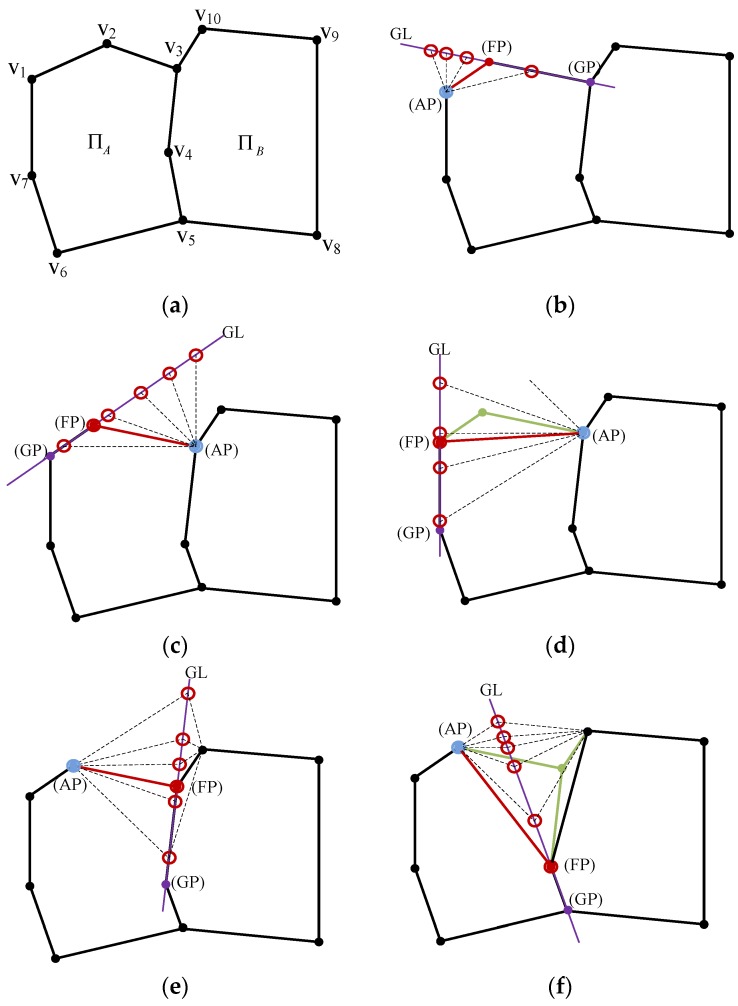
Examples of hypothesis generation (blue point: anchor point (*AP*), green point: removed point, purple point: guide point (*GP*), red point: floating point (*FP*), red circle: new possible positions of *FP*, red line: floating line (*FL*) and purple line: guide line (*GL*)): (**a**) initial configuration; (**b**) case 1; (**c**) case 2; (**d**) case 3; (**e**) case 4; and (**f**) case 5.

The hypothesis generation process proposes a set of possible hypotheses under certain configurations of a rooftop model (or building boundary). Suppose a rooftop model consists of a polygon $\Pi_{A}=\left\{v_{1},v_{2},v_{3},v_{4},v_{5},v_{6},v_{7}\right\}$ and a polygon $\Pi_{B}=\left\{v_{3},v_{4},v_{5},v_{8},v_{9},v_{10}\right\}$, where $v_{3}$, $v_{4}$ and $v_{5}$ are common vertices in both polygons (Figure 6a). A task is to generate possible hypotheses at a certain vertex considering a given configuration of rooftop model. The hypothesis generation process starts by defining an Anchor Point (*AP*), Floating Point (*FP*), and Guide Point (*GP*) and then by deriving a Floating Line (*FL* = [*AP*, *FP*]) and Guiding Line (*GL* = [*GP*, *FP*]). The role of *AP* is to define the origin of a line to be changed (*FL*). *FP* is a point to be moved while *GP* is used to generate *GL* which guides the movement of *FP*. Hypotheses are generated by moving *FP* along the *GL* with *AP* as an origin of *FL*. The orientation of *FL* is determined by representative angles of CLF which consists of eight directions as shown in Figure 5. There are different cases for hypothesis generation: (1) depending on a relative direction of *AP* and *FP* (forward (clockwise) and backward (anti-clockwise)); (2) depending on whether a vertex is removed (removal or non-removal); and (3) depending on whether *FP* is a common vertex in more than two adjacent polygons (common vertex or non-common vertex). For the reader's understanding, some cases are explained as follows:

- Case 1 (forward, non-removal, and non-common vertex): As shown in Figure 6b, $v_{1}$ and $v_{2}$ are assigned as *AP* (blue circle) and *FP* (red point), respectively. Hypotheses are generated by moving *FP* along to the *GL* where red circles represent new possible positions of $v_{2}$.
- Case 2 (backward, non-removal, and non-common vertex): As shown in Figure 6c, $v_{3}$ and $v_{2}$ are assigned as *AP* and *FP*, respectively. In contrast to case 1, *FP* is located in backward direction of *AP*.
- Case 3 (backward, removal, and non-common vertex): As shown in Figure 6d, after removing $v_{2}$ (green point), $v_{3}$ and $v_{1}$ are assigned as *AP* and *FP*, respectively. New hypotheses are generated by moving $v_{1}$.
- Case 4 (forward, non-removal, common vertex): As shown in Figure 6e, $v_{2}$ and $v_{3}$ are assigned as *AP* and *FP*, respectively. $v_{3}$ is a common vertex in $\Pi_{A}$ and $\Pi_{B}$. Because the position of $v_{3}$ changes, shapes of both polygons are changed.
- Case 5 (forward, removal, common vertex): As shown in Figure 6f, $v_{2}$ and $v_{4}$ are assigned as *AP* and *FP*, respectively. After $v_{3}$ is removed, $v_{4}$ is assigned as *FP* so that the position of $v_{4}$ is changed.

## 4. Parameter Optimization

In the MDL-based objective function, two types of weight parameters are used to evaluate the relative importance of sub-terms. One is a weight parameter (λ) for balancing the model closeness and the model complexity in Equation (1). The other is weight parameters ($W_{v},W_{d},W_{∠\theta }$) for the three sub-terms in the complexity term in Equation (6). In previous research [11], these weight parameters were set as constant values, which were empirically determined, for all building models (λ = 0.5 and $W_{v}=W_{d}=W_{∠\theta }=1$). However, buildings have different shapes and sizes in reality. In addition, the density of LiDAR points varies on data acquisition settings and flight height. These properties, which vary on individual buildings, may cause unbalanced values in model closeness and model complexity. For instance, when building shape is very simple and the number of observations is significantly large, the closeness value is relatively larger than the complexity value. As a result, optimization process may be dominant to the variation of the model closeness. Thus, the weight parameters have to be appropriately tuned in an automated manner by individually considering the properties of each building. To automatically determine proper weight values, we propose two different weighting methods: (1) Min-Max weighting method (Section 4.1); and (2) Entropy-based weighting method (Section 4.2). The Min-Max weighting method is used to balance the model closeness and the model complexity while the Entropy-based weighting method is employed to determine the weight values for the three sub-terms in the complexity term.

### 4.1. Min-Max Weighting Method

**Figure 7:**
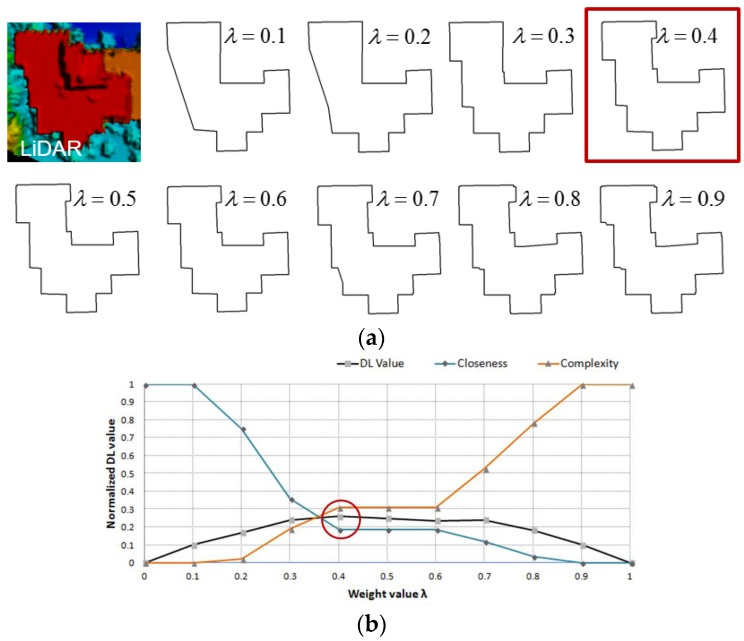
Min-Max based parameter determination: (**a**) optimal rooftop model for each λ value; and (**b**) corresponding normalized *DL* values where 0.4 is selected as the best λ value.

The proposed MDL-based objective function consists of two conflicting terms: the model closeness term ℒ(D|H) and the model complexity term ℒ(H) as shown in Equation (1). λ is a weight parameter which affects modeling result. The smaller the value of λ, the simpler the optimal model is. In contrast, a larger value of λ emphasizes goodness-of-fit to data, causing under-simplified model (or over-fitting problem) (see Figure 7). To automatically estimate an appropriate weight value, we adopt Min-Max criterion [40], which minimizes possible loss while maximizing the gain. In this study, the Min-Max principle is closely related to minimizing the cost value *DL* for each λ and maximizing contributions from both of two terms, thereby finding the optimal $\lambda^{*}\in \left[0,1\right]$. For each term, this leads to avoid the best scenario where one of two terms dominates by having an excessively low or high value of λ. To achieve this goal, the “Min” operator first finds the optimal model for each λ using Equation (2). Considering the boundary conditions, ℒ(H) at λ=0 and ℒ(D|H) at λ=1 corresponds to zero. Then, ℒ(D|H) and ℒ(H) are normalized using min-max normalization method, respectively, as follows:

(7) $$z_{i}=\frac{x_{i}-min\left(x\right)}{max\left(x\right)-min\left(x\right)}$$

where $z_{i}$ is a normalized value for the *i*th variable $x_{i}$; min(x) and max(x) are the minimum value and maximum value for variable *x*. After the total *DL* value is computed from normalized ℒ(D|H) and ℒ(H) for each λ, the “Max” operator derives an optimal weight value $\lambda^{*}$ by selecting the worst scenario showing the maximum *DL*. Figure 7 shows an example of the Min-Max weighting method. As shown in Figure 7a, as λ is close to 0, a simple model is selected as the optimal model. As λ. gets larger, the optimal model is more complex because the *DL* value is more affected by the closeness term. In this example, 0.4 is selected as the best λ because it produces the maximum *DL* value.

### 4.2. Entropy-Based Weighting Method

Prior to determining the weight parameter λ, we estimate the weight values of geometric parameters forming the complexity term ℒ(H) in Equation (6). The ℒ(H) consists of three geometric terms including the number of vertices, the number of identical line directions and the inner angle transition.

In multi-attribute decision making, an entropy weighting method, which is one of the objective methods, is used to determine appropriate weights for attributes [41]: the greater the value of the entropy corresponding to a special attribute, the smaller attribute’s weight. We adopt the entropy weighting method to determine the relative importance of three geometric terms in Equation (6). In information theory, entropy is understood as a measure of uncertainty about attributes drawn from data and can be normally characterized as follows:

(8) $$E\left(X\right)=-\overset{n}{\underset{i=1}{\sum }}p\left(x_{i}\right)\log_{2}\;p\left(x_{i}\right)$$

The basic formulation can be rewritten to calculate entropy in the existence of two possibilities *p* and *q* = 1 − *p* as follows:

(9) $$E=-\left(p\log_{2}\;p+q\log_{2}\;q\right)$$

where *p* represents the event that a current hypothesized parameter set belongs to a class of optimal model parameters and *q* indicates the reverse situation of *p*. In this study, a probability for each term in Equation (6) is derived by calculating a probability that each geometric factor in a given model can converge to the optimal model. The optimal model in terms of model complexity, according to the definition of model complexity discussed in Section 3.1, is represented by a rectangle where the number of vertices is four, the number of identical line directions is two, and all inner angles have no penalty. Thus, the probability that four vertices are randomly selected from $N_{v}$ vertices is one over four combinations of $N_{v}$, $p\left(v\right)=1/C_{4}^{N_{v}}$. Similarly, the probability that two identical line directions are selected from $N_{d}$ identical line directions is one over two combinations of $N_{d}$, $p\left(d\right)=1/C_{2}^{N_{d}}$. The probability of inner angle with no penalty in Equation (5) is 3/16. Because all inner angles have no penalty to be optimal model, the probability for $N_{∠\theta }$ is $p\left(∠\theta \right)=N_{v}\times 3/16$. The estimated probabilities are converted into entropy using Equation (9). Weight parameters for three sub-terms are determined as suggested in previous studies [41,42]:

(10) $$W_{v}=\frac{1-E\left(v\right)}{3-\left(E\left(v\right)+E\left(d\right)+E\left(∠\theta \right)\right)},\;W_{d}=\frac{1-E\left(d\right)}{3-\left(E\left(v\right)+E\left(d\right)+E\left(∠\theta \right)\right)},\;W_{∠\theta }=\frac{1-E\left(∠\theta \right)}{3-\left(E\left(v\right)+E\left(d\right)+E\left(∠\theta \right)\right)}$$

## 5. Results and Discussion

### 5.1. Data

**Figure 8:**
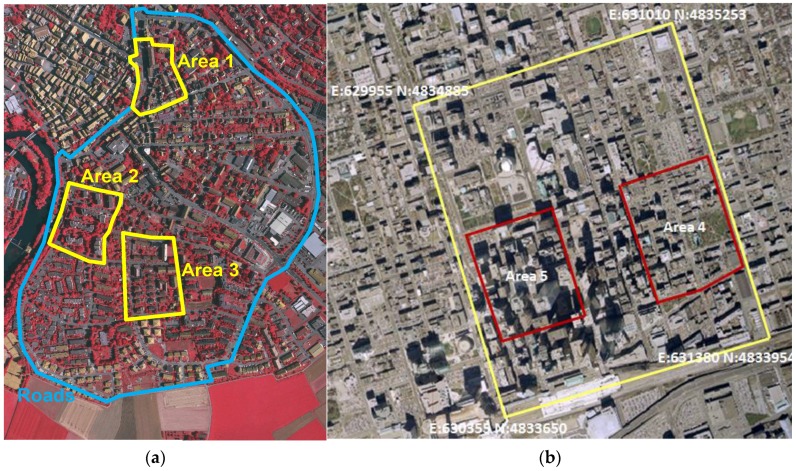
Test datasets: (**a**) Vaihingen (Area 1, Area 2, and Area 3); and (**b**) downtown Toronto (Area 4 and Area 5).

The performance of the proposed method was evaluated over the ISPRS benchmark datasets provided by the ISPRS WGIII/4 [43]. The ISPRS benchmark datasets consist of three sub-regions (Area 1, Area 2, and Area 3) of the Vaihingen dataset, and two sub-regions (Area 4 and Area 5) of the Toronto dataset (Figure 8). The Vaihingen dataset was acquired by Leica ALS50 system at an altitude of 500 m above ground level in August 2008. Ten strips are overlapped with 30% rate and an average point density is approximately 6.7/m^2^ (~0.39 m point spacing). The 3D positional accuracy shows approximately ±10 cm. The Vaihingen dataset contains typical European building types showing various shapes including gable, hip roof, and their mixed structures. The Toronto dataset was taken by Optech’s ALTM-ORION M system at an altitude of 650 m in 2009. The test area includes six strips with about 6/m^2^ average point density (~0.41 m point spacing). The dataset contains representative scene characteristics of a modern mega city in North America including a mixture of low- and high-story building and a complex cluster of high-rise buildings. For both datasets, reference building models were generated by manual stereo plotting method. More detailed explanation on the data can be found in [43]. To extract the building points, we applied the classification method described in [44].

### 5.2. Evaluation Metric

The ISPRS benchmark project on urban classification and 3D building modeling led by ISPRS WGIII/4 provides evaluation metrics to estimate the results obtained from the latest state-of-the-art algorithms for building detection and 3D building reconstruction [2]. The ISPRS evaluation metrics were designed for measuring the performance characteristics of individual algorithms by comparing multiple evaluation indices including confusion matrix (area-based and object-based), topological analysis among roof planes, and geometric accuracy (RMSE). Thus, the ISPRS metrics are used to evaluate our proposed method. In addition, we added two shape similarity measures (Hausdorff distance and turning function distance) and an angle-based evaluation index to evaluate different aspects of reconstructed building models. Hausdorff measures shape similarity between reference models and algorithmic models by taking the maximum distance among the minimum distances measured between each vertex for two model datasets [45]. In contrast to RMSE, which assesses the average difference between two models, the Hausdorff distance can measure the maximum shape difference caused by over-simplification and under-simplification without any pre-defined value for the proximity criterion. The turning function distance represents a cumulative measure of the angles through which a polygonal curve turns [46]. A turning function distance enables the direct measuring of turning pattern similarity between reference and algorithmic models. Thus, the turn function distance can measure a resemblance between two models at global scale. Additionally, an angle-based evaluation index measures the difference between main orientation of a building modeled in a reference dataset and the results produced by an algorithm. The main orientation of a building model is determined by analyzing the frequency of building lines for eight direction zones generated by the CLF. Table 1 summarizes an evaluation indices used in this paper.

### 5.3. Confusion Matrix-Based Evaluation

Evaluations using confusion matrix were applied under three different conditions: (a) by applying area-based method for outer building boundary; and by applying object-based method (b) for all roof planes; and (c) for roof planes with more than 10 m^2^, respectively (Table 2).

In the area-based evaluation (Table 2a), our proposed rooftop reconstruction algorithm showed that the completeness, correctness, and quality of the reconstructed building models are 91.5%, 97.4%, and 89.2%, respectively. The results indicate that most of resulting building models were properly overlapped to the corresponding reference building models. The error rate for the completeness is larger than the error rate for the correctness. This is due to the fact that most of the boundary points from irregularly distributed points are not exactly located on the real building outline but they often feature a small offset to it. The inexact observations cause boundary displacement which is generally positioned toward the inside of the building. As a result, a building model tends to be shrunken compared to the reference building model. This leads to the increase of *FNs* and the decrease of *TPs*, degenerating the completeness.

**Figure 9:**
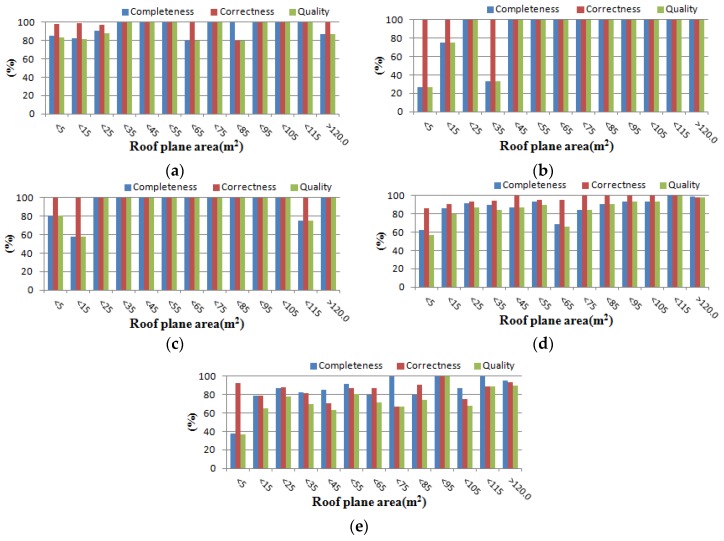
Object-based evaluation as a function of the roof plane area: (**a**) Area 1; (**b**) Area 2; (**c**) Area 3; (**d**) Area 4; and (**e**) Area 5.

In the object-based evaluation methods, a roof plane in one dataset was considered to be a true positive if a certain minimum percentage of its area (50% overlap) is covered by a roof plane in the other dataset. While the completeness, correctness, and quality for all roof planes are 79.5%, 96.0%, and 77.3%, respectively (Table 2b), the values are increased to 93.8%, 96.9%, and 91.3% if only large roof planes (>10 m^2^) are considered (Table 2c). The results indicate that small roof planes were not detected as well by our proposed method. This is mainly caused by the small number of points on small building roof planes which made it difficult to extract sufficient modeling cues for reconstructing rooftop models. Figure 9 clearly shows the effect of the size of roof plane. When only roof planes with an area smaller than 5 m^2^, are considered, the completeness is considerably low for all five datasets. In particular, the completeness for Area 2 (Figure 9b) and Area 5 (Figure 9e) were 26.3% and 37.4%, respectively. We observed that buildings in the two regions have many small objects on their roofs which were represented in reference building rooftop models.

As shown in Table 2, the area-based evaluations show that similar levels of model quality were achieved for both the Vaihigen dataset and the Toronto dataset. However, the object-based evaluations indicate that the model quality for the Vaihingen dataset is better than one for the Toronto dataset. This is mainly related to segmentation errors which occur more in complex scenes. We observed that many roof planes in the Toronto dataset were under-segmented by merging adjacent clusters. As a result, building rooftop models generated from under-segmented clusters caused a low success rate of the completeness.

In addition, we compared the evaluation results with those assessed for other algorithms that were reported in [2] where area-based evaluation results were not reported (Table 3). The object-based evaluation results (Table 3a) demonstrate that our method can outperform other building reconstruction algorithms except for the BNU in terms of the completeness and quality. In particular, when roof planes, whose area is larger than 10 m^2^, were considered, our proposed method showed more accurate results. The BNU, which outperform our method, was assessed only for Area 3. With regard to robustness, our proposed method outperforms the BNU. The correctness of our method is better than the average of all other evaluated methods. Considering that the correctness is above 90% for all compared methods except MON and FIE, the correctness of our method is large enough. In addition, the superiority of our method can be proven by Toronto dataset which consists of complex buildings. Only three participants submitted their results for Toronto dataset, and our method achieved the best results for all indices.

### 5.4. Shape-Based and Angle-Based Evaluations

Geometrical errors in planimetry, and in height were assessed using RMSE. The RMSE measures Euclidean distance in two different ways: (1) from a vertex in the reconstructed rooftop model to its closest vertex in reference model; and (2) from a vertex in the reference model to its closest vertex in the reconstructed rooftop model. Note that only distances shorter than a certain tolerance distance (<3 m) were considered as introduced by [2].

**Figure 10:**
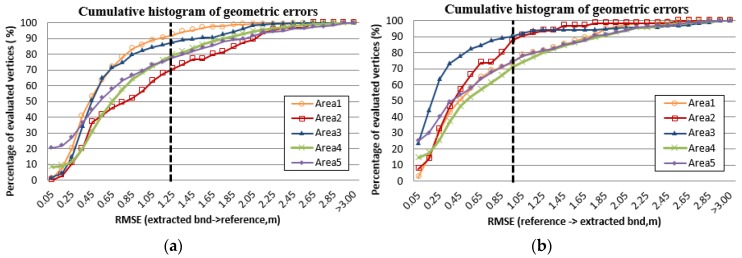
The cumulative histogram of geometrical errors: (**a**) RMSE of extracted vertices with respect to reference vertices; and (**b**) RMSE of reference vertices with respect to extracted vertices.

The average RMSE of distances in planimetry for the Vaihigen dataset and the Toronto dataset are 0.76 m and 0.96 m, respectively. As shown in Table 3b, the geometric accuracy is better than the average geometric accuracy of building models reconstructed by other algorithms. Figure 10 shows the cumulative histogram of geometric accuracy in RMSE over the five sub-regions. Overall, more than 70% of evaluated vertices are located with less than 1.25 m RMSE. In most test regions, the results of RMSE of reference vertices (Figure 10b) are better than those of RMSE of extracted vertices (Figure 10a). The reason is that the proposed method provides under-simplified models with redundant vertices (i.e., having more numbers of vertices compared to the reference model). Note that the closest vertex within a certain tolerance distance (>3 m) was used to calculated RMSE. Thus, RMSE of extracted vertices, which have redundant vertices, tends to be worse than one of reference vertices.

**Figure 11:**
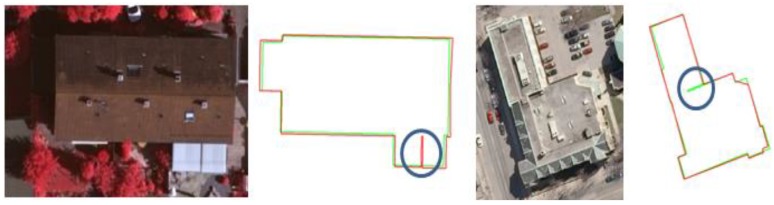
Examples of a large amount of Hausdorff distance for 2D outer boundary (Red: Reference, Green: extracted rooftop model).

Hausdorff distance was applied to 2D outer boundaries and to 3D roof planes with *1:1* correspondence, respectively (Table 4b). The averages of Hausdorff distance for 2D outer boundaries and for 3D roof planes are 1.81 m and 1.17 m, respectively. The results show that the maximum distance between the vertices of reference rooftop models and extracted rooftop models is expected to be less than roughly twice the RMSE by our proposed method. In addition, the average of the Hausdorff distance for 2D outer boundaries is larger than the value for 3D roof planes. This is mainly caused by topology relations between roof planes. As shown in Figure 11, two roof planes, which share a common edge in reference models (or in extracted models), were represented by separated roof planes in extracted models (or reference models). The different topology relations caused a large amount of shape differences in outer boundary representation.

**Figure 12:**
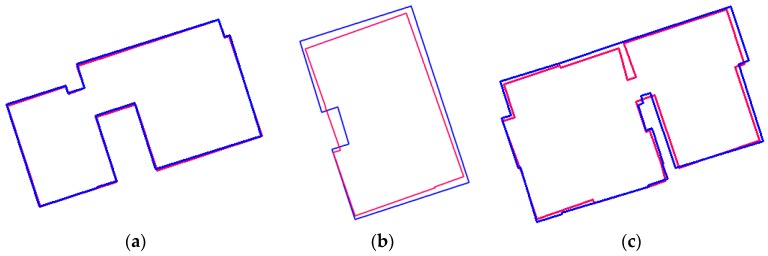
Approximate ranges of turning function distance (blue: reference, red: extracted model): (**a**) 0.016; (**b**) 0.055; and (**c**) 0.105.

Turning function distance, which measures how similar two shapes are, was applied to outer building boundaries and to roof planes with 90% overlap, respectively. Roughly, when the value is smaller than approximately 0.03, two corresponding shapes are very similar in terms of visual inspection. However, when the value is larger than approximately 0.05, the shapes are considerably dissimilar (Figure 12). For five sub-regions, the average turning function distances are 0.042 for 2D outer boundaries and 0.033 for 3D roof planes, respectively (Table 4c). Although turning function distances do not provide a specific range for which value is acceptable for building rooftop models, our results can be compared with examples given in Figure 12. The comparison indicates that the building rooftop models reconstructed by the proposed method can achieve acceptable shape similarities compared with reference building rooftop models in terms of visual inspection. Similarly to the results of Hausdorff distance, the turning function distance for 2D outer boundaries is larger than one for 3D roof planes due to different topologies and representations of rooftop models.

To evaluate the quality of model orientation, the angle difference was measured by calculating the difference of dominant orientations between reconstructed rooftop models and reference rooftop models. Table 4a shows the angle differences for five sub-regions where the averages of angle differences are 1.17° for 2D outer boundaries and 0.91° for 3D roof planes, respectively. Note that main angles for outer boundary and for 3D roof planes can be different because the main angle is separately determined for outer boundary and 3D roof planes. The orientation error was entirely caused by representative angles of CLF which were used to represent a regular pattern of the line orientation. The representative angles of CLF were calculated from all initial boundary lines connecting boundary points of individual building models without any prior knowledge of building orientations. Thus, a large amount of orientation error in small building models can be accidently caused if angles of the boundary lines were distorted by local distributions of boundary points.

Additionally, topology relations were assessed by comparing overlap area between reference rooftop planes and extracted rooftop planes. Table 5 represents the number of instances of *1:1*, *1:M*, *N:1*, and *N:M* relations. More than 63% of roof planes are matched with *1:1* relations; 22% of roof planes have *N:1* relations; 7% of roof planes have *1:M* relations; and 8% of roof planes have *N:M* relations. The topology errors are mainly caused by incorrect segmentation and incomplete modeling cues. In particular, relatively higher *N:1* relations are caused by under-segmentations and superstructures on roofs which often occur in complex scene. Thus, the *N:1* relations were observed more in the Toronto dataset.

### 5.5. Effect on Weight Parameters

**Figure 13:**
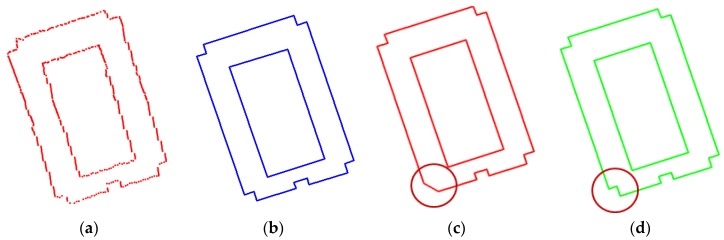
Effect on flexible weight parameters: (**a**) boundary points; (**b**) reference building model; (**c**) building model generated with fixed weight parameters; and (**d**) building model generated with flexible weight parameters.

To evaluate an effect of weight parameters in MDL-based objective function, we compared building models generated using fixed weight parameters with building models generated using the proposed weighting methods. Area-based evaluations using confusion matrix and shape-based indices were applied. The area-based evaluations using confusion matrix show an increase of 1.3% for the completeness, a decrease of 0.7% for the correctness, and an increase of 0.6% for the quality when the proposed weighting methods were used (Table 6). For Hausdorff distance and turning function distance, the improvements of 0.44 m and 0.003 were achieved, respectively (Table 7). While evaluation results using confusion matrix and evaluation results for turning function distance show slight improvements, the results for Hausdorff distance show relatively large improvements for all sub-regions except for Area 3. In addition, the most improvements for all evaluation methods were achieved by Area 4 where a relatively large number of shape differences at local scale between extracted models and reference models were observed. Figure 13 shows an example where shape difference at local scale is reduced by the proposed weighting methods. When fixed weight parameters were used, a lower part of the building model (red circle) was under-simplified (Figure 13c). This is related to the number of boundary points and a degree of model complexity. A large number of observations produced relatively high closeness value compared with complexity value. This caused imbalance between two values because fixed weight parameters do not consider the property of an individual building model. In contrast, the closeness term and the complexity term were balanced by using flexible weight parameters (Figure 13d). As shown in Table 6 and Table 7 and Figure 13, applying flexible weight values makes positive effects in preserving shapes similar to reference rooftop models.

### 5.6. Visual Inspection

**Figure 14:**
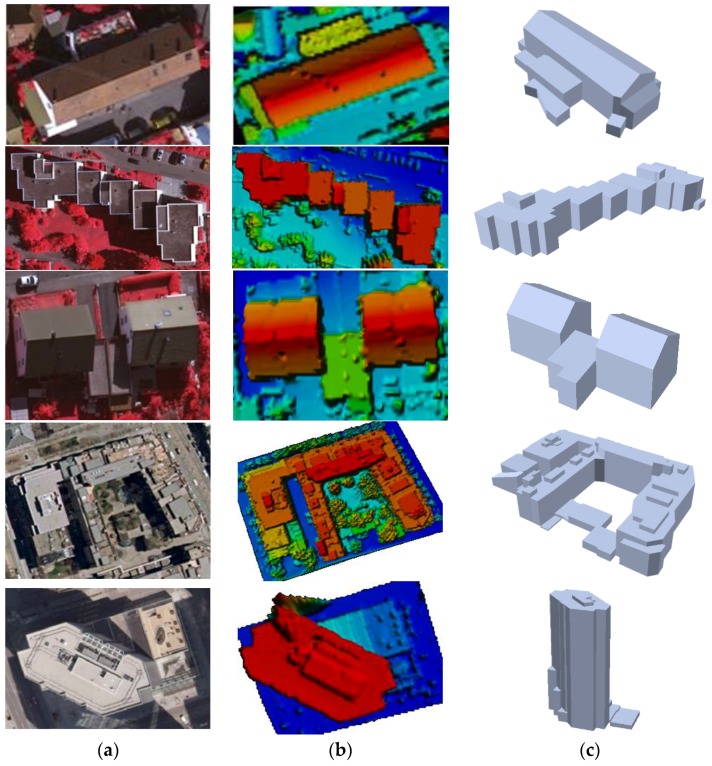
Reconstructed building models with complex roof structure: (**a**) image; (**b**) LiDAR point clouds; and (**c**) perspective view of the reconstructed 3D building model.

**Figure 15:**
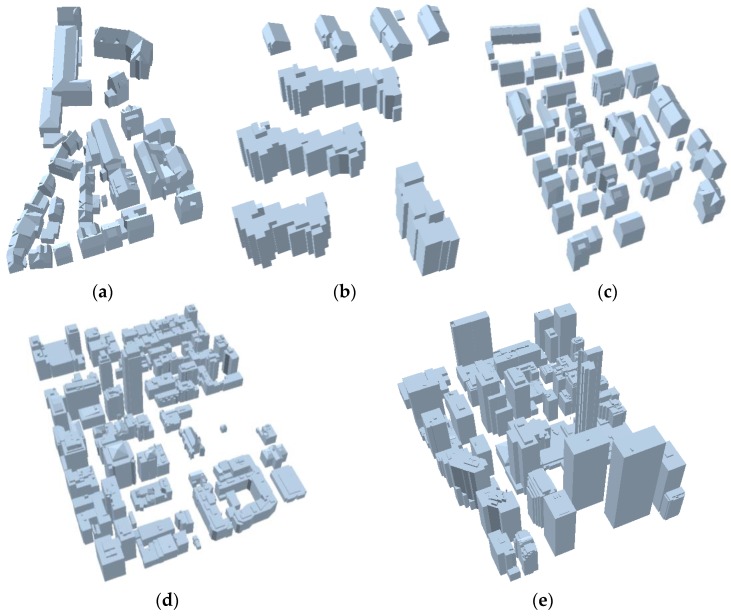
Reconstructed building models: (**a**) Area 1; (**b**) Area 2; (**c**) Area 3; (**d**) Area 4; and (**e**) Area 5.

Figure 14 visualizes reconstructed building rooftop models which are representative buildings of five sub-regions. Visual inspection indicates that the proposed building reconstruction method can robustly provide accurate regularized 3D building rooftop models in both simple scenes and complex scenes. Figure 15 shows all reconstruction building rooftop models over our test datasets.

## 6. Conclusions

In this study, we proposed an automatic 3D building reconstruction method which covers a full chain of rooftop modeling. Building-labeled points were segmented into homogeneous clusters with a hierarchical structure, which enables explicit interpretation of building rooftop configuration. To effectively gather evidence of a rooftop structure, three linear modeling cues including intersection line, step lines, and boundaries were separately extracted by considering their characteristics. In the proposed method, regularization is the most important process, which implicitly imposes geometric regularities on reconstructed rooftop models based on MDL principle. In the MDL framework, finding a regularized rooftop model was recognized as a model selection problem. The best model was selected by minimizing *DL* values among competing hypotheses generated by a newly designed hypothesis generation process. To automatically control weight parameters, a Min-Max based weighting method and Entropy-based weighting method were proposed. The experimental results showed that the proposed method can provide qualitatively and quantitatively well-regularized 3D building rooftop models. More specifically, the results are summarized as follows:

- The proposed method provided a robust solution for 3D rooftop modeling regardless of scene complexity, e.g., typical European style structure with relatively simple building shapes as well as complex clusters of high-rise buildings. This is achieved by the hierarchical clustering of building rooftop points. Even though modeling cues were incompletely extracted, the BSP method produced geometrically and topologically correct rooftop models.
- Evaluation results using confusion matrix showed that the proposed method outperforms other building reconstruction algorithms. However, object-based evaluation results indicated that our method has a limitation on extracting small size rooftops. It is a common problem in data-driven approaches due to the fact it is difficult to extract modeling cues from the small number of roof points. One possible solution for this problem is to combine the data-driven method and model-driven method by taking their complementary properties.
- The proposed weighting methods have a positive effect on the building regularization process. Results for Hausdorff distance showed that the values are considerably improved when flexible weight parameters in MDL objective function were applied. In particular, shape deformation (under-simplified or over-simplified model) at a local scale was reduced by the proposed method.
- Angle based evaluation shows that the method has 1.17° difference compared to the reference. However, the main orientations of building models in this study were determined without any prior knowledge. Thus, the accidently large amount of orientation error can occur in small size buildings. One possible solution for the problem is to use image data, which can explicitly provide the orientation of building model.

In current study, 3D point clouds obtained by airborne LiDAR was used as a primary information source for the automation of reconstructing rooftop models. However, the proposed method can be also applicable to photogrammetric point clouds generated by various dense matching technologies [53]. As future work, we will investigate the impact of photogrammetric point clouds the quality of 3D rooftop models reconstructed, and thus seek for an optimal solution to make the proposed method be robust to various quality of point clouds. In addition, it will be required to examine the impact of the accuracy of building detection, especially in relation to the occlusion caused by the presence of vegetation and adjacent buildings.

## Figures and Tables

**Table 1:** Performance evaluation metric.

Evaluation Index		Description	Object to Be Evaluated
Completeness, Correctness, Quality	Area-based	Completeness, correctness, and quality determined on a per-area level	Building outer-boundary
	Object-based	Completeness, correctness, and quality determined on a per-roof-plane level	Roof planes
*N_1:1_*, *N_1:M_*, *N_N:1_*, *N_N:M_*		Difference in the topologies of the extracted roof planes and the reference	Roof planes
RMSE x, y, z		Geometrical errors in planimetry and in height; only distance shorter than 3 m are considered	vertices
Hausdorff distance		Evaluation for partly deformed shape	Building outer-boundary; Plane with 1:1 correspondence
Turning function distance		Evaluation for entire shape similarity	Building outer-boundary; Plane with 1:1 correspondence
Angle-based index		Difference in main angle of building model between reference and resulting rooftop models	Building outer-boundary; Plane with 1:1 correspondence

**Table 2:** Confusion matrix-based evaluations.

Dataset	Sub-Set	# of Building	# of Plane	(a) Area-Based Evaluation			Object-Based Evaluation					
							(b) All Roof Planes			(c) Roof Planes (10 m^2^ Area)		
				Comp. (%)	Corr. (%)	Quality (%)	Comp. (%)	Corr. (%)	Quality (%)	Comp. (%)	Corr. (%)	Quality (%)
Vaihingen	Area 1	38	288	90.6	98.8	89.6	88.9	98.2	87.5	93.9	98.5	92.6
	Area 2	15	69	91.3	99.7	91.0	73.9	100	73.9	95.8	100	95.8
	Area 3	57	235	88.6	99.7	88.4	86.4	100	86.4	97.6	100	97.6
	Sub-total	110	592	90.2	99.4	89.7	83.1	99.4	82.6	95.8	99.5	95.3
Toronto	Area 4	58	967	93.7	96.9	90.9	82.1	94.8	78.6	92.4	96.2	89.2
	Area 5	38	640	93.1	92.0	86.1	66.1	87.1	60.2	89.5	89.6	81.1
	Sub-total	96	1607	93.4	94.5	88.5	74.1	91.0	69.4	91.0	92.9	85.2
Total		206	2199	91.5	97.4	89.2	79.5	96.0	77.3	93.8	96.9	91.3

**Table 3:** Evaluation results of algorithms reported in Rottensteiner et al. (2014).

Dataset	Algorithm	(a) Object-based evaluation using confusion matrix						(b) RMSE_XY (m)
		For All Roof Planes			For roof Planes (10 m^2^ Area)			
		Comp. (%)	Corr. (%)	Quality (%)	Comp. (%)	Corr. (%)	Quality (%)	
Vaihingen	MON [47]	77.5	89.7	71.2	90.3	91.4	83.5	0.90
	VSK [23]	74.2	98.6	73.5	86.1	98.6	85.2	0.83
	ITCE1 [26,48]	69.4	90.1	63.1	78.4	90.3	69.5	1.00
	ITCE2 [26,48]	69.8	98.3	68.7	76.8	100.0	76.8	1.03
	ITCX1 [49]	69.5	98.1	68.7	74.4	98.0	73.2	0.70
	ITCX2 [49]	82.0	92.9	76.8	91.0	98.1	89.3	0.70
	ITCX3 [49]	82.8	94.9	78.7	93.2	97.8	91.2	0.70
	CAS [43]	68.5	100.0	68.5	81.2	100.0	81.2	0.75
	TUD [50]	70.0	95.8	67.8	78.8	98.6	78.0	0.70
	YOR [11]	79.9	99.5	79.5	91.8	99.7	91.6	0.63
	KNTU [43]	80.4	96.7	78.3	91.9	97.7	90.0	0.90
	FIE [51]	82.6	83.1	70.7	88.7	93.4	83.5	1.10
	CKU [25]	82.1	96.8	80.1	91.4	99.4	90.9	0.73
	BNU [52]	87.2	100.0	87.2	96.0	100.0	97.1	0.60
	Proposed method	83.1	99.4	82.6	95.8	99.5	95.3	0.76
Toronto	YOR [11]	70.0	91.7	66.2	86.4	92.1	80.4	0.90
	CKU [25]	69.5	81.8	60.1	79.1	81.4	67.1	1.75
	FIE [51]	82.3	91.5	49.9	60.4	91.9	57.3	1.40
	Proposed method	74.1	91.0	69.4	91.0	92.9	85.2	0.96

**Table 4:** Angle-based and shape-based evaluations.

Dataset	Sub-Set	For 2D Outer Boundary			For 3D Roof Planes with *1:1* Correspondence (90% Overlap)		
		(a) Angle Difference (deg)	(b) Hausdorff Distance (m)	(c) Turning Function Distance	(a) Angle Difference (deg)	(b) Hausdorff Distance (m)	(c) Turning Function Distance
Vaihingen	Area 1	1.32	1.33	0.049	0.78	0.46	0.020
	Area 2	1.62	1.26	0.040	1.11	1.77	0.041
	Area 3	0.59	0.93	0.031	0.44	0.48	0.016
	Sub-total	1.18	1.17	0.040	0.78	0.90	0.026
Toronto	Area 4	1.30	2.44	0.046	1.30	1.38	0.040
	Area 5	1.04	3.10	0.046	0.91	1.75	0.047
	Sub-total	1.17	2.77	0.046	1.11	1.57	0.044
Total		1.17	1.81	0.042	0.91	1.17	0.033

**Table 5:** Topology evaluation.

Dataset	Sub-Set	Topology (Reference Rooftop Planes: Extracted Rooftop Planes)			
		N*_1:1_*	N*_N:1_*	N*_1:M_*	N*_N:M_*
Vaihingen	Area 1	125	36	17	8
	Area 2	29	5	9	1
	Area 3	72	49	6	2
	Sub-total	226	90	32	11
Toronto	Area 4	300	89	32	47
	Area 5	147	52	6	33
	Sub-total	447	141	38	80
Total		673	231	70	91

**Table 6:** Effect on weight parameters in confusion matrix-based evaluation.

Dataset	Sub-Set	(a) Fixed Weight Parameters			(b) Weight Parameters Determined by the Proposed Method			(b)–(a)		
		Comp.	Corr.	Quality	Comp.	Corr.	Quality	Comp.	Corr.	Quality
Vaihingen	Area 1	88.8	99.5	88.4	90.6	98.8	89.6	+1.8	−0.7	+1.2
	Area 2	90.2	99.8	90.0	91.3	99.7	91.0	+1.1	−0.1	+1.0
	Area 3	88.8	99.7	88.5	88.6	99.7	88.4	−0.2	0.0	-0.1
	Sub-total	89.3	99.7	89.0	90.2	99.4	89.7	+0.9	−0.3	+0.7
Toronto	Area 4	89.5	98.2	88.1	93.7	96.9	90.9	+4.2	−1.3	+2.8
	Area 5	93.8	93.5	88.1	93.1	92.0	86.1	−0.7	−1.5	-2.0
	Sub-total	91.7	95.9	88.1	93.4	94.5	88.5	+1.8	−1.4	+0.4
Total		90.2	98.1	88.6	91.5	97.4	89.2	+1.3	−0.7	+0.6

**Table 7:** Effect on weight parameters in shape-based evaluation.

Dataset	Sub-Set	(a) Fixed Weight Parameters		(b) Weight Parameters Determined by the Proposed Method		(a)–(b)	
		Hausdorff Distance (m)	Turning Function Distance	Hausdorff Distance (m)	Turning Function Distance	Hausdorff Distance (m)	Turning Function Distance
Vaihingen	Area 1	1.44	0.047	1.33	0.049	0.11	−0.002
	Area 2	1.58	0.041	1.26	0.040	0.32	0.001
	Area 3	0.91	0.036	0.93	0.031	−0.02	0.005
	Sub-total	1.31	0.041	1.17	0.040	0.14	0.001
Toronto	Area 4	3.76	0.058	2.44	0.046	1.32	0.012
	Area 5	3.58	0.045	3.10	0.046	0.48	−0.001
	Sub-total	3.67	0.052	2.77	0.046	0.90	0.006
Total		2.25	0.045	1.81	0.042	0.44	0.003

## Supplementary Materials

The following are available online at www.mdpi.com/1424-8220/17/3/621/s1, Video S1: Sequential results of implicit regularization for 3D rooftop reconstruction.
